# Characteristics of patients with asthma overprescribed short-acting beta-agonist (SABA) reliever inhalers stratified by blood eosinophil count in North East London: a cross-sectional observational study

**DOI:** 10.3399/BJGPO.2023.0020

**Published:** 2023-05-31

**Authors:** Paul Pfeffer, Hajar Hajmohammadi, James Cole, Chris Griffiths, Sally Hull, Anna De Simoni

**Affiliations:** 1 Barts and The London School of Medicine and Dentistry, Queen Mary University of London, London, UK; 2 Wolfson Institute of Population Health, Queen Mary University of London, London, UK

**Keywords:** asthma, short-acting beta-agonist, albuterol, primary health care, general practice

## Abstract

**Background:**

Overprescription of short-acting beta-agonist (SABA) inhalers and blood eosinophil count have strong associations with exacerbation risk in asthma. However, in the authors' recent publication only a minority of patients overprescribed SABA (≥6 inhalers in 12 months) were eosinophilic (≥0.3 x 10^9^ cells/l).

**Aim:**

To compare the characteristics of eosinophilic and non-eosinophilic patients with asthma overprescribed SABA inhalers, and identify latent classes using clinical variables available in primary care.

**Design & setting:**

Cross-sectional analysis of patients with asthma in North East London, England, using primary care electronic health record data.

**Method:**

Unadjusted and adjusted multi-variate regression models and latent class analysis.

**Results:**

Eosinophilia was significantly less likely in female patients (*P* = 0.004), those with multiple mental health comorbidities (*P*<0.001), and those with SABA on repeat prescription (*P*<0.001). Latent class analysis identified the following three classes of patients overprescribed SABA: class 1, which represents classical uncontrolled asthma (oral steroids required for exacerbations, step 2–3 asthma medications, high probability of being eosinophilic); class 2, which represents mild asthma (low exacerbation frequency, low asthma medication step, low probability of being eosinophilic); and class 3, which represents difficult asthma (high exacerbation frequency despite high-strength preventer inhalers, low probability of being eosinophilic). The mild asthma class was the largest.

**Conclusion:**

Many patients being overprescribed SABA were non-eosinophilic with a low exacerbation frequency, suggesting disproportionately high SABA prescription compared with other asthma control markers. Potential reasons for high SABA prescription in these patients included repeat prescription (being dispensed but not taken) and use of SABA for non-asthma breathlessness (for example, breathing pattern disorders with anxiety). Further research is needed into management of SABA overuse in patients without other markers of uncontrolled asthma.

## How this fits in

Overuse of SABA reliever inhalers and peripheral blood eosinophilia are two risk factors both associated with increased risk of asthma exacerbations. Stepping-up inhaled corticosteroids is effective at reducing future exacerbations in eosinophilic patients; however, this study showed most patients with asthma who were overprescribed SABA inhalers were not eosinophilic.

The characteristics of eosinophilic and non-eosinophilic patients overprescribed SABA were found to be different. Lack of eosinophilia in a patient with asthma with high SABA use should alert the clinician to look for other causes of overuse, such as inappropriate SABA use for other causes of breathlessness.

Through analysis of routinely collected clinical data, distinct patient subgroups overusing SABA are readily identifiable by clinicians, potentially prompting different management approaches. More research is needed in this area.

## Introduction

Overuse of SABA inhalers, such as salbutamol relievers, is an acknowledged indicator of uncontrolled asthma with increased risk of exacerbations and hospital admissions,^1,2^ yet remains highly prevalent.^3,4^ Indeed SABA overuse can paradoxically worsen asthmatic airways pathology and dampen response to SABA taken appropriately when needed.^5,6^ Additionally, as most SABA inhalers are pressurised metred-dose inhalers (pMDIs), their use is associated with a major carbon footprint, which is harmful to the environment.^7^ There is therefore a current focus on reducing patient SABA overuse and overprescription.

For patients with frequent SABA use, most asthma guidelines recommend that the clinician addresses potential poor adherence to preventer medications and inhaler technique, and then considers a step-up in asthma medications including higher-dose inhaled corticosteroids.^8,9^ Such approaches encourage a one-size-fits-all model in contrast to increasing conceptual understanding of asthma as a disease of treatable traits, and that not all ‘asthma’ symptoms are due to uncontrolled small airways inflammation.^10^ While uncontrolled asthma is associated with increased SABA usage, it is less certain whether most patients who overuse SABA have uncontrolled asthma, in terms of active small airways inflammation.

Factors associated with SABA overprescription in the North East London population were investigated by the authors in a recent publication, and an association with prescription type was identified.^11^ The objective of this further evaluation was to examine whether the characteristics of patients overusing SABA differ by presence of an eosinophilia or not, and by potential latent class analysis of patients overprescribed SABA. Blood eosinophil counts, as a surrogate biomarker of airways type 2 (T2) inflammation, are increasingly recognised as a marker of exacerbation risk in asthma and an indicator of patients likely to benefit from increased inhaled corticosteroids.^12,13^ The findings suggest that there are different phenotypes of patients overusing SABA , who may need different approaches to manage their SABA overuse in primary care.

## Method

### Study population

Patients overprescribed SABA inhalers in the preceding 12 month period and with a full blood count measured in the past 2 years were selected from the previous evaluation of SABA prescription. Primary care data were used from more than 30 000 patients aged 5–80 years with asthma from North East London.^11^ Primary care data, including all prescriptions for inhaled asthma medications and courses of oral corticosteroids in the preceding year, were extracted on secure N3 terminals from EMIS Web during October and November 2021. Data extracted on SABA prescriptions included prescribing modality (acute or automatic, repeat dispensing, repeat prescribing). All participants had a coded diagnosis of asthma in their primary care electronic medical record. SABA overprescription was defined as the prescription of ≥6 salbutamol 200-dose 100 µg/dose equivalent inhalers in the preceding 12 months, as previously described.^11^ Potential selection bias was addressed by including all patients with asthma from all primary care practices in the region. Use of clinical data extracted from regional standard-of-care templates for clinical care addressed potential information bias.

### Outcome and determinant variables

Blood eosinophil counts, undertaken as part of clinically indicated full blood counts, were extracted from primary care medical records. Where patients had had more than one eosinophil count in the preceding 2 years, the count closest to the (evaluation) data extraction date was used. Characteristics were compared between those whose last eosinophil count was <0.3 × 10^9^cells/l (no eosinophilia) and those ≥0.3 x 10^9^ cells/l (eosinophilia).

Patient demographics (age, sex, ethnic group, body mass index [BMI], smoking history) were extracted as most recently recorded in primary care records. Ethnic categories were based on the 18 categories of the UK 2011 census and were combined into four groups reflecting the study population. Asthma medication step was extracted from the patient’s last annual asthma review. Courses of oral corticosteroids in the preceding year were determined from prescribing records. Medication prescription refill for inhaled corticosteroids was calculated from prescription data as previously described.^11^

Comorbidities data were extracted on 16 conditions that form part of the UK Quality and Outcomes Framework (QOF),^14^ using the earliest recorded diagnostic code before the start of the study, supplemented by SNOMED codes (www.snomed.org) for chronic rhinitis and generalised anxiety.

### Statistics

To compare the characteristics of eosinophilic and non-eosinophilic groups, regression models were undertaken, both unadjusted and adjusted for other variables of interest, as described below. Regression models were analysed in R (version 4.0.4).

For the latent class analysis, models were developed with blood eosinophil count, courses of oral corticosteroids in the preceding year, asthma medication step, and SABA prescription type as indicator variables and other variables as covariates. Models were developed with 2, 3, and 4 classes and then the best one was selected based on Bayesian information criterion (BIC). Models with smaller values of BIC were preferred, with the use of additional parameters justified by sufficient improvement in the likelihood of the fitted model. This criterion provided a balance between improvement in fit (represented by increased log-likelihood) and model complexity (represented by the number of parameters used). Based on the BIC values (55 394, 55 239, and 55 457 for 2, 3, and 4 classes), latent class analysis with three classes was selected. These analyses were run using package poLCA in R (version 4.0.4).

### Patient and public involvement

Input from Asthma UK Centre for Applied Research (AUKCAR) patient and public involvement (PPI) representatives has informed this programme of work addressing SABA overuse.^15^ AUKCAR PPI have reviewed the results of the evaluation of which this publication forms a part, with their views informing the analysis of the ongoing asthma programme.

### Data reporting and availability

This cross-sectional observational research was reported according to the STrengthening the Reporting of OBservational studies in Epidemiology (STROBE) guidelines. The data analysed formed part of a service evaluation intrinsic to a regional quality improvement project, and under this framework the dataset cannot be publicly released.

## Results

In the recent analysis of SABA prescribing in North East London, 10 081 of 30 694 patients with asthma were overprescribed SABA inhalers (≥6 more salbutamol 100 micrograms/dose [200-dose/inhaler] or equivalent inhalers in the preceding year). On their last blood eosinophil count, 3507 patients overprescribed SABA were eosinophilic and 4375 not eosinophilic (no full blood count for remaining 2199 patients).

In univariate analyses, there were significant differences by eosinophil count in patients overprescribed SABA in terms of patient characteristics and asthma medication usage. For example, there was an odds ratio (OR) of 0.85 for being eosinophilic compared with non-eosinophilic for those who had previously smoked referenced to those who had never smoked (*P* = 0.005). In multivariate analysis, factors significantly associated with being eosinophilic included Asian ethnic group (OR 1.78; 95% confidence interval [CI ] = 1.57 to 1.96; *P*<0.001); being on step two or three asthma medications (OR 1.28; 95% CI = 1.06 to 1.53; *P* = 0.009; and OR 1.30; 95% CI = 1.04 to 1.52; *P* = 0.017); and having received ≥3 courses of oral steroids in the preceding year (OR 1.20; 95% CI = 1.18 to 1.36; *P* = 0.041). Eosinophilia was significantly less likely in female patients (OR 0.88; 95% CI = 0.78 to 0.95; *P* = 0.004); those with multiple mental health comorbidities (OR 0.58; 95% CI = 0.41 to 0.78; *P*<0.001); or those for whom SABA was issued through repeat or repeat dispense prescription types (OR 0.81; 95% CI = 0.72 to 0.91; *P*<0.001; and OR 0.69; 95% CI = 0.50 to 0.94; *P* = 0.021; Table 1).

**Table 1.** Characteristics of patients with ≥6 short-acting beta-agonist (SABA) bronchodilator relievers prescribed over the preceding year stratified by last blood eosinophil count

Adult patients (aged ≥18 years)		Patients, *n*		Univariate OR	Multivariate OR(95% CI)
		Eos <0.3*n* = 4375	Eos ≥0.3*n* = 3507	Eos ≥0.3	Eos ≥0.3 × 10^9^ cells/l
**Age**	Adult (18–60 years)	3160	2591	ref	ref
	Older adult (>60 years)	1215	916	0.92	0.91 (0.81 to 1.02)
**Sex**	Male	1756	1521	ref	ref
	Female	2619	1986	0.88*	0.88* (0.78 to 0.95)
**BMI**	Healthy weight	859	679	ref	ref
	Underweight	65	39	0.76	0.83 (0.55 to 1.27)
	Overweight	1136	988	1.10	1.10 (0.95 to 1.25)
	Obese	1608	1305	1.03	1.12 (0.97 to 1.26)
	Unknown	707	496	0.89	0.97 (0.83 to 1.14)
**Ethnic group**	White	1881	1167	ref	ref
	Mixed	147	107	1.82*	0.88 (0.61 to 1.12)
	Asian or Asian British	1505	1714	1.84*	1.78* (1.57 to 1.96)
	Black	500	303	0.98	0.93 (0.79 to 1.10)
	Other or unclassified	342	216	1.02	0.99 (0.83 to 1.14)
**IMD score**	1 (least deprived)	600	458	ref	ref
	2	779	590	0.99	0.91 (0.76 to 1.07)
	3	973	804	1.08	0.93 (0.78 to 1.08)
	4	1035	878	1.11	0.99 (0.83 to 1.14)
	5 (most deprived)	988	777	1.03	0.95 (0.80 to 1.12)
**Smoking status**	Never smoked	2567	2166	ref	ref
	Currently smokes	829	632	0.90	1.03 (0.89 to 1.16)
	Formerly smoked	972	698	0.85*	0.96 (0.84 to 1.07)
	Unknown	7	11	1.86	2.34 (0.64 to 6.27)
**Asthma medication step**	Step 1	388	252	ref	ref
	Step 2	1689	1471	1.34*	1.28* (1.06 to 1.53)
	Step 3	952	830	1.34*	1.30* (1.04 to 1.52)
	Step 4 + Step 5	185	133	1.11	1.15 (0.80 to 1.44)
	Unknown	1161	821	1.09	1.15 (0.94 to 1.37)
**Oral steroid, *n***	0	3256	2,504	ref	ref
	1	596	526	1.15*	1.12 (1.00 to 1.30)
	2	209	196	1.22*	1.20* (1.12 to 1.33)
	≥ 3	314	281	1.16*	1.20* (1.18 to 1.36)
**Physical health** **(number of** **comorbidities)**	0	1110	806	ref	ref
	1	1499	1221	1.12*	1.10 (0.97 to 1.24)
	2–3	1385	1190	1.18*	1.11 (0.97 to 1.25)
	≥4	381	290	1.05	0.94 (0.76 to 1.13)
**Mental health** **(number of** **comorbidities)**	0	2468	2,146	ref	ref
	1	906	679	0.86*	0.90 (0.79 to 1.25)
	2	878	623	0.82*	0.89 (0.78 to 1.00)
	3	123	59	0.55*	0.58* (0.41 to 0.78)
**MPR category**	Zero	306	196	ref	ref
	Underuse	885	712	1.26*	1.17 (0.92 to 1.42)
	Reasonable use	2175	1,809	1.30*	1.19 (0.94 to 1.42)
	Overuse	1009	790	1.22*	1.11 (0.88 to 1.35)
**Prescription type**	Acute + automatic	737	707	ref	ref
	Repeat	3517	2725	0.81*	0.81* (0.72 to 0.91)
	Repeat dispensed	121	75	0.65*	0.69* (0.50 to 0.94)

BMI = body mass index. Eos = eosinophil count. IMD = Index of Multiple Deprivation. MPR = medication prescription refill rate (categories as previously defined).^11^

Odds ratios (OR) with *P* value significance <0.05 in bold with asterisk. Multivariate analyses include adjustment for all other factors listed.

Given the association with numbers of comorbidities, the authors further examined whether there might be associations with specific comorbidities (Table 2). Depression (*P* = 0.005), anxiety (*P* = 0.001), comorbid chronic obstructive pulmonary disease (COPD; *P* = 0.030), heart failure (*P* = 0.047), and gastro-oesophageal reflux disease (GORD; *P* = 0.047) were associated with significantly decreased OR of being eosinophilic. Rhinitis was associated with significantly increased OR (*P*<0.001).

**Table 2.** Comorbidities in patients with ≥6 short-acting beta-agonist (SABA) bronchodilator relievers over the preceding year stratified by last blood eosinophil count

Adult patients (aged ≥18 years)		Patients with SABA ≥6*, n*		Univariate OR	+ **adjustment**(95% CI)
		Eos <0.3*n* = 4375	Eos ≥0.3*n* = 3507	Eos ≥0.3	Eos ≥0.3×10^9^ cells/l
**Comorbidities**	Atrial fibrillation	93	58	0.77	0.80 (0.57 to 1.12)
	Cancer	161	106	0.82	0.85 (0.65 to 1.09)
	CHD	244	237	1.23*	1.09 (0.90 to 1.32)
	CKD	332	264	0.99	0.99 (0.83 to 1.17)
	COPD	15	5	0.42*	0.37* (0.12 to 0.97)
	Dementia	20	11	0.69	0.61 (0.28 to 1.28)
	Depression	1,330	902	0.79*	0.85* (0.77 to 0.94)
	Diabetes	963	843	1.12*	0.98 (0.88 to 1.09)
	Epilepsy	123	75	0.76*	0.78 (0.588 to 1.06)
	Heart failure	94	55	0.73	0.71* (0.50 to 1.00)
	Hypertension	1390	1100	0.98	0.96 (0.87 to 1.06)
	Learning disabilities	57	35	0.76	0.77 (0.49 to 1.17)
	Mental health	219	149	0.84	0.86 (0.69 to 1.07)
	Palliative care	26	16	0.77	0.69 (0.36 to 1.29)
	Peripheral arterial disease	29	22	0.95	0.94 (0.52 to 1.64)
	Stroke and TIA	109	78	0.89	0.90 (0.66 to 1.21)
	Anxiety	1482	1051	0.84*	0.89* (0.81 to 0.98)
	Gastro-oesophageal	846	633	0.92	0.85* (0.76 to 0.96)
	Rhinitis	1945	1799	1.32*	1.22* (1.12 to 1.34)

CHD = coronary heart disease. CKD = chronic kidney disease. COPD = chronic obstructive pulmonary disease. Eos = eosinophils. TIA = transient ischaemic attack

Odds ratios (OR) with *P* value significance <0.05 in bold with asterisk. Univariate OR, and multivariate OR after additional adjustment for sex, ethnic group, asthma medication step, oral steroid courses, and prescription type.

**Figure 1.**
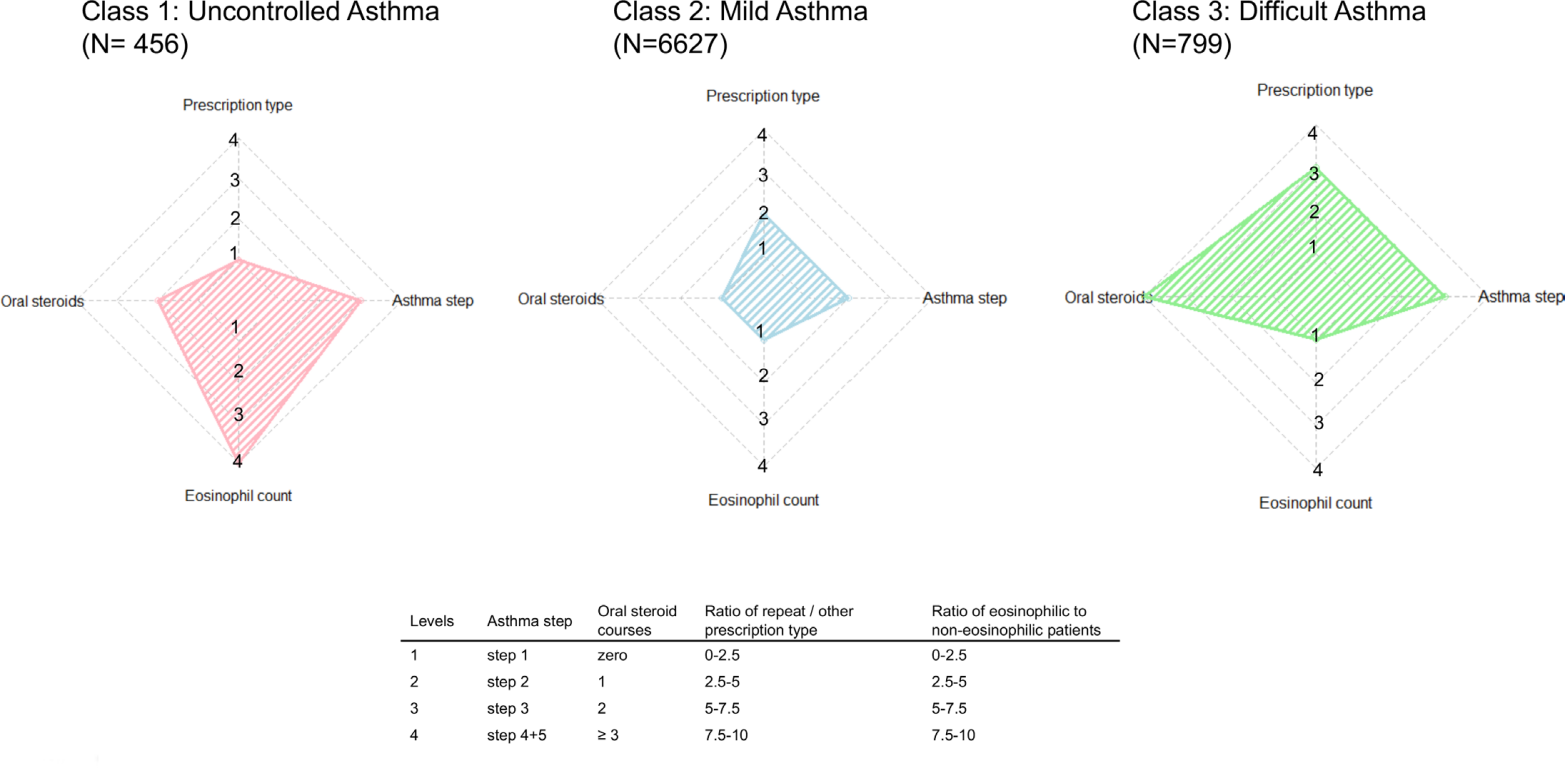
Characteristics of latent classes of patients overprescribed SABA. Radar plots for each class showing the asthma medication step and number of oral steroids courses in preceding year of highest probability for members of that class (horizontal positive and negative spokes); ratio of (probabilities for) members having SABA on repeat prescription versus other prescription type, and for an eosinophil count ≥0.3 versus<0.3 × 10^9^ cells/l (vertical positive and negative spokes). Variables in latent class analysis (LCA) analysed as categorical with levels for radar plot axis steps as per embedded table.

Given the significant differences in patients overprescribed SABA by eosinophil count, the authors next examined whether there might be latent subclasses of patients overprescribed SABA by latent class analysis. Indicator variables of blood eosinophil count, courses of oral corticosteroids in the preceding year, asthma medication step, and SABA prescription type were chosen (Figure 1; Supplemental Table S1, Figure S1). A latent class analysis model with three classes was selected in preference to models with two or four classes based on BIC values.

Class 1 comprised 456 patients and was relatively enriched for patients with an eosinophilia, ≥1 courses of oral steroids in the preceding year, and step 2 or 3 asthma medications. Classes 2 and 3, relative to class 1, had lower probabilities for patients to be eosinophilic (in both, the probability of a patient being eosinophilic was <50%) and higher probabilities for patients having a repeat prescription type for their SABA inhalers.

Class 2 comprised 6627 patients compared with 799 patients in class 3. Comparing class 2 and class 3, those in class 3 had much higher probabilities for having had courses of oral steroids in the preceding year despite patients in class 3 having a higher probability of being on higher asthma step medications than patients in class 2.

## Discussion

### Summary

There are significant differences in the characteristics of eosinophilic and non-eosinophilic patients with asthma overprescribed SABA inhalers in the North East London population, with the majority not eosinophilic on their last full blood count. Adjusted multivariate regression models found eosinophilia in patients overprescribed SABA to be associated with male sex, Asian ethnic group, multiple courses of oral steroids in the preceding year, SABA not of repeat or repeat dispensing prescription modality, and absence of multiple mental health comorbidities. In terms of specific comorbidities, eosinophilia was positively associated with rhinitis, and negatively associated with anxiety, depression, GORD, cardiac failure, and comorbid COPD. To further explore these differences, a latent class analysis was conducted with indicator variables of blood eosinophil count, courses of oral corticosteroids in the preceding year, asthma medication step, and SABA prescription type. This identified three latent classes that corresponded, as discussed below, to patients with classical eosinophilic uncontrolled asthma (class 1), those with difficult asthma (class 3) and a class of patients with ‘mild’ asthma with high SABA prescription discordant to other measures of asthma control (class 2).

### Strengths and limitations

The strength of this analysis is the large size of the population studied and use of routine electronic health record data from primary care, reducing potential selection bias. Furthermore, use of routine clinical data, together with the latent class methodology in particular, revealed patient subgroups readily identifiable by clinicians in routine primary care practice, potentially prompting different management approaches.

A limitation of this analysis is that not all of the patients overprescribed SABA from the original evaluation could be included, as a minority of patients did not have a blood eosinophil count within the required timeframe. Although the relative sizes of the three latent classes identified may have been affected by that issue, it is unlikely that inclusion of ‘missing’ patients would have changed the characteristics of the identified latent classes. Although the relative proportions of patients within each latent class may vary geographically with differing healthcare systems and prescribing preferences, the three identified latent classes requiring different interventions are likely to be generalisable to other primary care asthma populations.

### Comparison with existing literature

The associations identified in the regression studies are consistent with those reported in other populations. For example, the association between mental health comorbidities and SABA overprescription in non-eosinophilic patients is consistent with previous studies showing an association between SABA overuse and mental health conditions,^16^ but the finding has been extended to show this association is a particular feature in non-eosinophilic patients. The association between eosinophilia and frequent exacerbations is well-described in poorly controlled asthma.^10,12,13^ The higher prevalence of eosinophilia in Asian patients with poorly controlled asthma in this evaluation is consistent with reports of differences by ethnic group in characteristics of patients with severe asthma in the UK.^17^ Differences by sex have also been described in severe asthma, consistent with the findings in a broader population of patients with asthma in this analysis. For example, Eastwood and colleagues have recently reported poor symptom control with discordantly low biomarker levels is particularly a feature in female patients with severe asthma.^18^

The latent class analysis extends the published literature by identifying that the subgroup of eosinophilic patients with exacerbating asthma is a minority of the larger population of patients with asthma overprescribed SABA. Latent class analysis is a form of cluster analysis that can include categorical variables and is designed to identify latent clusters within a population. Cluster analyses have been conducted in asthma research before, but predominantly in severe asthma. Haldar *et al* did include a minority of primary care-managed patients with asthma in their cluster analysis of predominantly patients with severe asthma, and interestingly reported a symptomatic female-predominant cluster with absent eosinophilic airway inflammation, a cluster with concordant symptoms and eosinophilic airway inflammation, and a cluster with few symptoms and absent eosinophilia.^19^ The present research extends the understanding of SABA overprescription in patients with asthma by describing subgroups easily identifiable by routine clinical markers in primary care, including blood eosinophil count, and these have significant implications for clinical practice, as described below.

### Implications for research and practice

The three latent classes identified correspond to distinct patient groups for which different approaches are required in primary care if their SABA overprescription is to be safely addressed. Class 1 in the latent class analysis was enriched for patients with eosinophilia with uncontrolled asthma, who would likely respond to guideline-based review and increase in inhaled corticosteroids in primary care. Therefore, this class has been termed classical uncontrolled asthma. In these patients switching to a combined anti-inflammatory reliever may be a pragmatic approach to ensuring adequate inhaled corticosteroids to prevent future exacerbations.^20,21^ However, these patients with classical uncontrolled asthma, who are the focus of most guidelines, were a minority of the entire population of patients overprescribed SABA.

On the other side, many of the patients in class 3 had been prescribed multiple courses of oral steroids despite being on higher-step asthma medications, identifying them as patients who may benefit from referral to specialist severe asthma services, as recommended in most guidelines. Therefore, this class has been termed difficult asthma.^22^ Many patients in class 3 were not eosinophilic and potentially their symptoms and exacerbations might be secondary to other causes than asthma; for example, inducible laryngeal obstruction and breathing pattern disorders.^23^ However, the addition of tezepelumab to the biologics armamentarium has extended the range of patients with severe asthma who benefit from biologics to include those without current eosinophilia or other raised T2 biomarker, and the lack of raised inflammatory biomarkers should not dissuade primary care clinicians from referring these patients to regional difficult asthma services.^24^

Although a single eosinophil count cannot exclude uncontrolled T2 airways inflammation, the high proportion of patients who were not eosinophilic suggests many patients were being overprescribed SABA, despite controlled airway inflammation and low exacerbation risk as exemplified by patients in class 2. This class has been termed mild asthma given the low medication step for most of these patients and low exacerbation frequency (despite discordant high SABA prescription).

The majority of patients in class 2 were receiving their SABA inhalers under repeat prescription (or repeat dispensing) and may be receiving unwanted repeat prescriptions of SABA inhalers they are not using. Switching SABA prescription type for these patients to as-requested acute prescriptions may significantly reduce overprescription, and addressing SABA prescription type should be included in future asthma guidelines.

However, approximately 20% of patients with mild asthma (class 2) were receiving multiple SABA inhalers through acute prescriptions, suggesting actual overuse despite a low proportion of these patients being eosinophilic. SABA inhalers may be being taken inappropriately for other causes of breathlessness, such as breathing pattern disorders and anxiety; this possibility needs further research. Stepping up the strength of their inhaled corticosteroids is unlikely to be beneficial in these non-eosinophilic patients and may be associated with unwarranted inhaled corticosteroid side effects. Alternative strategies for managing symptoms in these patients are needed.^25,26^ How best to manage such patients is not a focus of most asthma guidelines, despite these patients being in the majority in the latent class analysis. Importantly, frequent usage of SABA for complex breathlessness is likely associated with extra-pulmonary side effects of excess ß-adrenergic stimulation in addition to the complications of not actually treating the underlying pathology driving the breathlessness.^27,28^

Research is now needed on how to reduce SABA usage in those patients overusing despite control of underlying asthmatic airways inflammation. SABA reduction and/or withdrawal needs to be done with a safe approach but also in a manner that respects the complex health beliefs of many patients who overuse SABA inhalers.^29,30^ Elements of an inpatient SABA withdrawal programme have been described,^31^ but given the numbers of patients overusing SABA, consideration needs to be given to how this can be safely done in the community.

In conclusion, there are significant characteristic differences among patients with asthma overprescribed SABA between those who are and are not eosinophilic. In a latent class analysis, only a minority in the largest class of patients (class 2) were eosinophilic on their last blood eosinophil count. The lack of eosinophilia in many patients overprescribed SABA raises concern that these patients are either collecting but not using the inhalers, or inappropriately using SABA inhalers for other causes of breathlessness. Research and guidelines are now needed on how to manage SABA overuse in patients inappropriately taking excessive doses despite controlled airways inflammation.

## Data

This cross-sectional observational research was reported according to the STrengthening the Reporting of OBservational studies in Epidemiology (STROBE) guidelines. The data analysed formed part of a service evaluation intrinsic to a regional quality improvement project, and under this framework the dataset cannot be publicly released.
